# Construction of a risk prediction model for Alzheimer’s disease in the elderly population

**DOI:** 10.1186/s12883-021-02276-8

**Published:** 2021-07-07

**Authors:** Lingling Wang, Ping Li, Ming Hou, Xiumin Zhang, Xiaolin Cao, Hongyan Li

**Affiliations:** 1 Department of Neurology, People’s Hospital of Xinjiang Uygur Autonomous Region, NO.91 Tianchi Road, Tianshan District, Urumqi, Xinjiang, 830001 Uygur Autonomous Region China; 2 Department of Nursing, People’s Hospital of Xinjiang Uygur Autonomous Region, Uygur Autonomous Region, Xinjiang, 830001 China

**Keywords:** Alzheimer’s disease, Risk factor, Prediction model, Nomogram, Diagnosis

## Abstract

**Background:**

Dementia is one of the greatest global health and social care challenges of the twenty-first century. The etiology and pathogenesis of Alzheimer’s disease (AD) as the most common type of dementia remain unknown. In this study, a simple nomogram was drawn to predict the risk of AD in the elderly population.

**Methods:**

Nine variables affecting the risk of AD were obtained from 1099 elderly people through clinical data and questionnaires. Least Absolute Shrinkage Selection Operator (LASSO) regression analysis was used to select the best predictor variables, and multivariate logistic regression analysis was used to construct the prediction model. In this study, a graphic tool including 9 predictor variables (nomogram-see precise definition in the text) was drawn to predict the risk of AD in the elderly population. In addition, calibration diagram, receiver operating characteristic (ROC) curve and decision curve analysis (DCA) were used to verify the model.

**Results:**

Six predictors namely sex, age, economic status, health status, lifestyle and genetic risk were identified by LASSO regression analysis of nine variables (body mass index, marital status and education level were excluded). The area under the ROC curve in the training set was 0.822, while that in the validation set was 0.801, suggesting that the model built with these 6 predictors showed moderate predictive ability. The DCA curve indicated that a nomogram could be applied clinically if the risk threshold was between 30 and 40% (30 to 42% in the validation set).

**Conclusion:**

The inclusion of sex, age, economic status, health status, lifestyle and genetic risk into the risk prediction nomogram could improve the ability of the prediction model to predict AD risk in the elderly patients.

**Supplementary Information:**

The online version contains supplementary material available at 10.1186/s12883-021-02276-8.

## Introduction

Alzheimer’s disease (AD) is a neurodegenerative disease that mainly occurs in the elderly and is the most common cause of dementia [1]. More than 90% of AD cases occur in people over 65 [2]. With the aging of world population, the prevalence of AD is on the rise. The prevalence of dementia in people aged ≥60 years worldwide is reported to be between 5 and 7% [3]. Therefore, accurate identification of individuals at high risk of dementia is particularly important for early diagnosis and intervention.

Significant progress has been made in terms of risk factors for AD. For example, numerous studies have shown that risk factors in early years (education), middle age (hypertension, obesity, hearing loss, traumatic brain injury and alcohol abuse) and later years (smoking, depression, physical inactivity, social isolation, diabetes and air pollution) may contribute to an increased risk of dementia [4–6]. Higher levels of childhood education and lifetime education are associated with a lower risk of dementia [7]. Both genetic and lifestyle factors are vital in determining the individual risk of developing AD and other subtypes of dementia [8]. There is growing evidence that avoiding smoking, physical activity, moderate alcohol consumption and a healthy diet reduce the risk of developing dementia [9–13]. Based on the above factors, we can identify high-risk groups for AD and carry out targeted disease prevention measures, but there has been no recognized good risk assessment tool.

Multiple studies have demonstrated that nomogram is a novel risk prediction model combining multiple indicators rather than univariate analysis based on multivariate logistic analysis, which is important for screening and clinical practice [14–16]. Nomogram is currently widely used for risk prediction of various diseases, including hypertension [17], stroke [18], etc. The application of the model can accurately screen relevant variables and indicators, and determine the most appropriate risk factors. A previous study [19] constructed a nomogram map to predict the probability of conversion from mild cognitive impairment (MCI) to AD. This study combined neuroimaging features, cerebrospinal fluid (CSF) biomarkers and clinical assessment to play a significant role in clinical diagnosis and prediction. In this study, we constructed a risk prediction model for AD in the elderly by collecting clinical data and combining with questionnaire data.

## Materials and methods

### Data collection

Based on a previous research results [20], we finally determined sex, age, body mass index (BMI), marital status, education level, economic status, health status (whether suffering from midlife high blood pressure, diabetes, herpesvirus infection, stroke, traumatic brain injury, depression, etc.), lifestyle (including smoking, exercise, diet, alcohol) and genetic risk (if there is a family history of dementia) as the nine risk factors. A total of 555 medical records of elderly patients with AD previously diagnosed in our hospital were collected between October 2018 and December 2019, and 544 elderly patients without AD in this region were investigated. The demographic characteristics including the abovementioned 9 risk factors of all participants were acquired by questionnaire. The study was approved by the Medical Ethics Committee of People’s Hospital of Xinjiang Uygur Autonomous Region, and all participants were informed and signed written consent forms.

### Inclusion and exclusion criteria

Inclusion criteria: (1) According to the National Institute on Aging and the Alzheimer’s Association (NIA-AA), the diagnostic criteria for AD were as follows: clinically identified dementia, which was recorded by mini-mental state examination, blessed dementia rating scale, or similar test, and confirmed by a neuropsychological test; deficits in 2 or more domains of cognition; progressive deterioration of memory and other cognitive functions; no disturbance of consciousness; age of onset ranging from 40 to 90 years old, most commonly after 65 years old; no systemic disease or other brain diseases, which could explain the progressive deficits in memory and cognition [21]. (2) Patients were ≥ 60 years old (since most dementia events occur in the elderly) and had lived in the region for at least 6 months or permanently. Exclusion criteria: (1) Basic information of patients was not available due to cognitive impairment and/or inability to participate independently in the cohort. (2) There were serious organic diseases, such as tumors, major surgery, etc.

### Grading criteria

There are four types of marital status: unmarried, married (first marriage with a spouse, digamy with a spouse, remarriage with a spouse), widowed and divorced. Patients were rated based on current marital status, with 1 representing unmarried, 2 representing widowed or divorced, and 3 representing married with a spouse. The education levels were divided into high (university degree or other professional qualification), middle (high school or junior high school), and low (practical qualification related to work). Economic status was divided into five categories based on the Townsend Deprivation Index (which combines information on social class, employment, cars, housing, etc). Higher scores indicate better marital status, higher education levels and better economic status, respectively.

Health status was evaluated based on current disease information, and diseases such as midlife hypertension, diabetes, herpesvirus infection, stroke, traumatic brain injury and depression were considered comprehensively. The criteria were as follows: one point for having 5 or more diseases, 2 points for having 3 or 4 diseases, 3 points for having 2 or 3 diseases, 4 points for having 1 disease and 5 points for not having any disease. Higher scores represent better health status.

The lifestyle score was based on four established risk factors for dementia (smoking status, physical activity, diet and alcohol consumption). Smoking status was classified as current smoking or non-smoking. Regular physical activity was defined as at least 150 min of moderate exercise per week or 75 min of vigorous activity per week. A healthy diet is based on recommendations for cardiometabolic health that focus on eating at least four of seven commonly consumed foods, which are often associated with better later cognition and a reduced risk of dementia. Moderate alcohol was defined as 0 to 14 g/d for women and 0 to 28 g/d for men. Lifestyle scores ranged from 1 to 5, with a higher score indicating greater adherence to a healthy lifestyle. As for genetic risk, 1 score was for not clear, 2 for no family history of dementia and 3 for family history of dementia.

### Statistical analysis

R 3.6.1 [22] software was used for statistical analysis. First, 1099 participants were randomly divided into a training set (824 participants) and a validation set (275 participants) at a ratio of 3:1 using the R “caret” package [23]. “glmnet” package [24] was used to run least absolute shrinkage and selection operator (LASSO) regression analysis, which is a contraction and variable selection method for linear regression models. In order to obtain a subset of predictor variables, LASSO regression analysis shrinks the regression coefficient of some variables to zero by imposing constraints on model parameters, thus minimizing the prediction error of quantitative response variables. Variables with zero regression coefficients were excluded from the model after contraction, while variables with non-zero regression coefficients were selected as the most correlated with response variables. We set family = “binomial””, which applies to the binary discrete dependent variable, considering the dependent variable as AD or not (0/1). Then we set type.measure = “deviance”, that was −2log-likelihood. Based on −2log-likelihood and binary discrete dependent variables, LASSO regression analysis in R software was used to centralize and normalize the contained variables for k-fold (usually 10-fold) cross-validation, and then the best Lambda value was selected. The model provided by Lambda.lse has good performance, but with the fewest number of independent variables. Therefore, the LASSO method was used to analyze data in the training set to select the best predictors of dementia, including sex, age, BMI, marital status, education level, economic status, health status, lifestyle and genetic risk. The above included variables were used for preliminary screening of risk factor variables.

Then, we used the “rms” package [25] of R language to carry out logistic regression. By introducing the features selected in the LASSO regression model, we used multivariate logistic regression analysis to construct the prediction model. Key features included odds ratios (OR), 95% confidence intervals (CI), and *p* values. Statistically significant predictors in both groups were selected to establish the AD risk prediction model and a nomogram prediction model was developed using the rms package of R language. In addition, several validation methods were used to estimate the accuracy of the risk prediction model by using the data in the training set and the validation set. We used R language “pROC” package [26] for receiver operating characteristic curve (ROC). The area under the curve (AUC) was used to identify the quality of the nomogram to distinguish true positive from false positive. We used the “rms” package to draw and calculate the calibration curve for evaluating the calibration of AD risk nomogram, accompanied by the Hosmer-Lemeshow test (HLtest.R). The “rmda” package [27] was used for decision curve analysis (DCA) to determine the clinical utility of nomogram in this population based on the net benefit of different threshold probabilities.

## Results

### Basic characteristics of participants

The study included 1099 participants with an average age of 66.85 ± 4.07 years, of whom 555 had AD and 544 were non-demented subjects. All participants were randomly divided into the training set (*n* = 824) and the validation set (*n* = 275) at a ratio of 3:1. The basic characteristics of all participants were shown in Table 1.

**Table 1:** Baseline characteristics of all participants

Characteristics	Participants (***n*** = 1099)	With AD (***n*** = 555)	Without AD (***n*** = 544)	Training set (*n* = 824)	Validation set (***n*** = 275)	***P-***value
**Age (year)**	66.85 ± 4.07	67.06 ± 4.23	66.62 ± 3.88	66.82 ± 4.01	66.94 ± 4.23	0.047
**Sex, n (%)**						0.039
Male	711 (64.70)	362 (65.23)	349 (64.15)	543 (65.9)	168 (61.09)	
Female	388 (35.30)	193 (34.77)	195 (35.85)	281 (34.1)	107 (38.91)	
**BMI (kg/m**^**2**^**)**	23.14 ± 2.84	23.19 ± 2.91	23.15 ± 2.65	23.17 ± 2.86	23.06 ± 2.78	0.605
**Marital status, n (%)**						0.302
1	360 (32.76)	179 (32.25)	181 (33.27)	266 (32.28)	94 (34.18)	
2	349 (31.76)	166 (29.91)	183 (33.64)	264 (32.04)	85 (30.91)	
3	390 (35.49)	210 (37.84)	180 (33.09)	294 (35.68)	96 (34.91)	
**Education level, n (%)**						0.051
1	454 (41.31)	257 (46.31)	197 (36.21)	340 (41.26)	114 (41.45)	
2	205 (18.65)	64 (11.53)	141 (25.92)	153 (18.57)	52 (18.91)	
3	440 (40.04)	234 (42.16)	206 (37.87)	331 (40.17)	109 (39.64)	
**Economic status, n (%)**						< 0.001
1	336 (30.57)	209 (37.66)	127 (23.35)	253 (30.7)	83 (30.18)	
2	203 (18.47)	97 (17.48)	106 (19.49)	152 (18.45)	51 (18.55)	
3	172 (15.65)	79 (14.23)	93 (17.1)	133 (16.14)	39 (14.18)	
4	160 (14.56)	67 (12.07)	93 (17.1)	117 (14.2)	43 (15.64)	
5	228 (20.75)	103 (18.56)	125 (22.98)	169 (20.51)	59 (21.45)	
**Lifestyle, n (%)**						0.038
1	359 (32.67)	193 (34.77)	166 (30.51)	266 (32.28)	93 (33.82)	
2	349 (31.76)	166 (29.91)	183 (33.64)	264 (32.04)	85 (30.91)	
3	153 (13.92)	78 (14.05)	75 (13.79)	114 (13.83)	39 (14.18)	
4	146 (13.28)	86 (15.5)	60 (11.03)	112 (13.59)	34 (12.36)	
5	92 (8.37)	32 (5.77)	60 (11.03)	68 (8.25)	24 (8.73)	
**Health status, n (%)**						0.025
1	294 (26.75)	163 (29.37)	131 (24.08)	216 (26.21)	78 (28.36)	
2	258 (23.48)	131 (23.6)	127 (23.35)	193 (23.42)	65 (23.64)	
3	213 (19.38)	107 (19.28)	106 (19.49)	158 (19.17)	55 (20)	
4	188 (17.11)	91 (16.4)	97 (17.83)	145 (17.6)	43 (15.64)	
5	146 (13.28)	63 (11.35)	83 (15.26)	112 (13.59)	34 (12.36)	
**Genetic risk, n (%)**						0.019
1	151 (13.74)	77 (13.87)	74 (13.6)	117 (14.2)	34 (12.36)	
2	482 (43.86)	220 (39.64)	262 (48.16)	362 (43.93)	120 (43.64)	
3	466 (42.40)	258 (46.49)	208 (38.24)	345 (41.87)	121 (44)	

### Independent risk factors in the training set

**Fig. 1:**
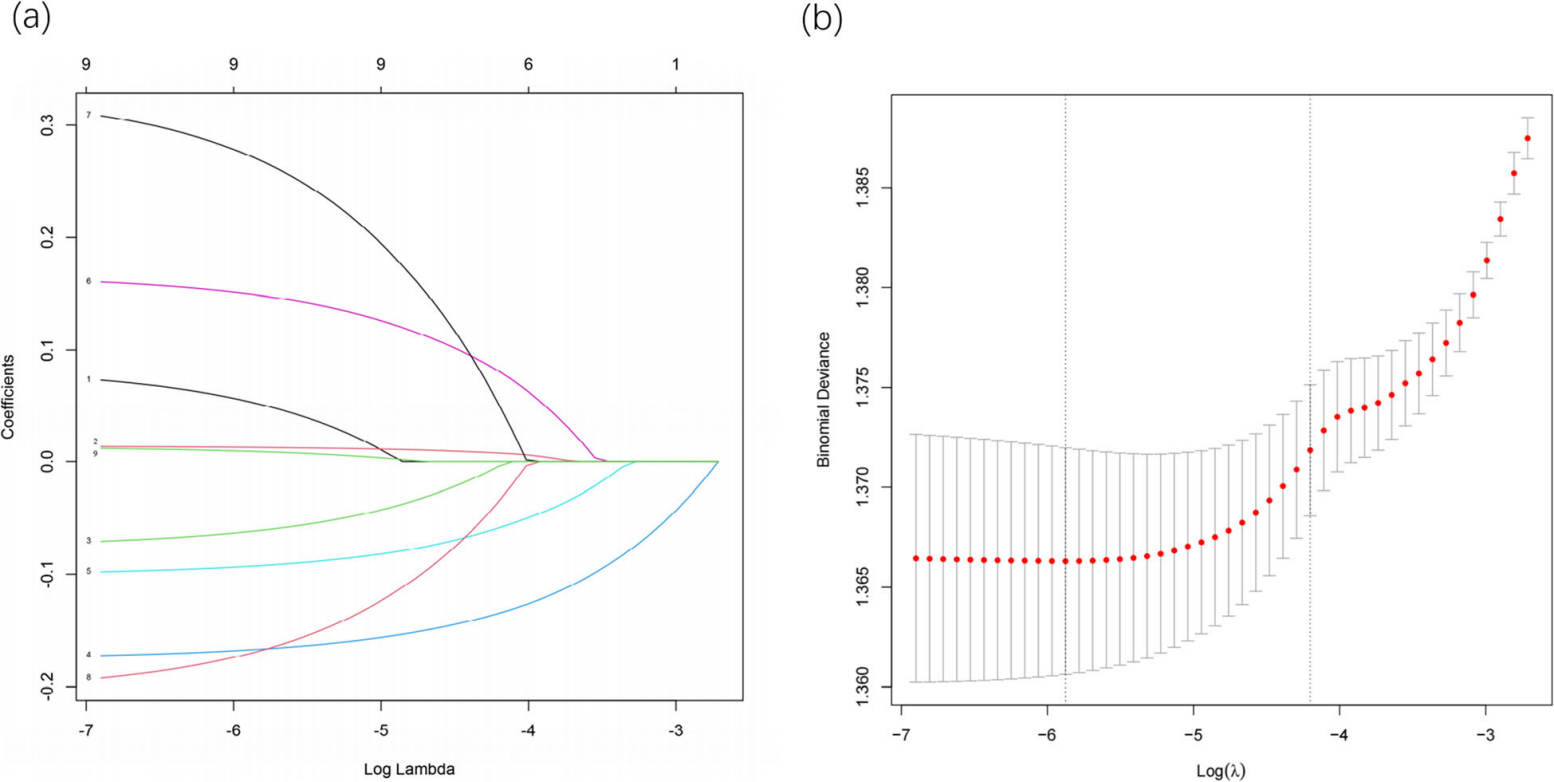
Selection of variables by LASSO binary logistic regression model and construction of coefficient distribution map according to log (lambda) sequence. **a** By deducing the best lambda, six variables with non-zero coefficients were selected; **b** After verifying the best parameter (lambda) in the LASSO model, a partial likelihood deviance (binomial deviance) curve was plotted versus log (lambda), and a vertical dotted line was plotted with 1 standard error

Multivariate logistic regression analysis showed that sex, age, economic status, health status, lifestyle and genetic risk were risk factors for AD in the elderly population we studied (Fig. 1).

### Prediction model construction

LASSO regression analysis was used to select the predictor variables from Table 1, and multivariate logistic regression was used to establish the prediction model. Six of the original nine variables were included in the risk prediction model, namely sex, age, economic status, health status, lifestyle and genetic risk. These six variables had non-zero coefficients in the LASSO regression model. The prediction model was represented by a nomogram and it was used for quantitative prediction of the risk probability of developing AD in the elderly population.

**Fig. 2:**
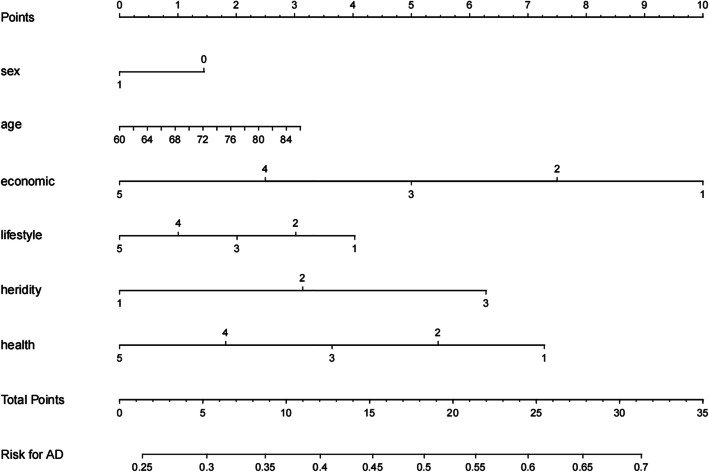
Risk prediction model of AD in the elderly (nomogram) sex: 1 presents male; 0 presents female

The logistic regression analysis results of these 6 variables were listed in Table 2. Since there were significant statistical differences among these six predictors, they were introduced into the prediction model to develop the AD risk nomogram (Fig. 2). For example, by using the nomogram model, it could be concluded that a 63-year-old man, male, in moderate economic condition and good health, without other diseases, enjoying smoking and drinking, with regular exercises and normal diet, having no genetic risk, had a 33.4% risk of developing AD.

**Table 2:** Logistic regression analysis of risk predictors for AD in the elderly

Intercept and variables	coefficient	Z value	*P-value*	Odds Ratio	95CI%
Intercept	−0.8911	−0.82			
Sex	0.033	0.26	*0.023*	1.034	0.804–1.329
Age	0.0205	1.34	*0.019*	1.086	0.963–1.224
Economic	−0.1756	−4.35	*0.012*	0.591	0.466–0.749
Lifestyle	−0.0988	−2.21	*0.034*	0.743	0.572–0.967
Heredity	0.1702	1.92	*0.004*	1.405	0.993–1.989
Health	−0.0525	−1.09	*0.001*	0.901	0.745–1.088

### Prediction model verification

**Fig. 3:**
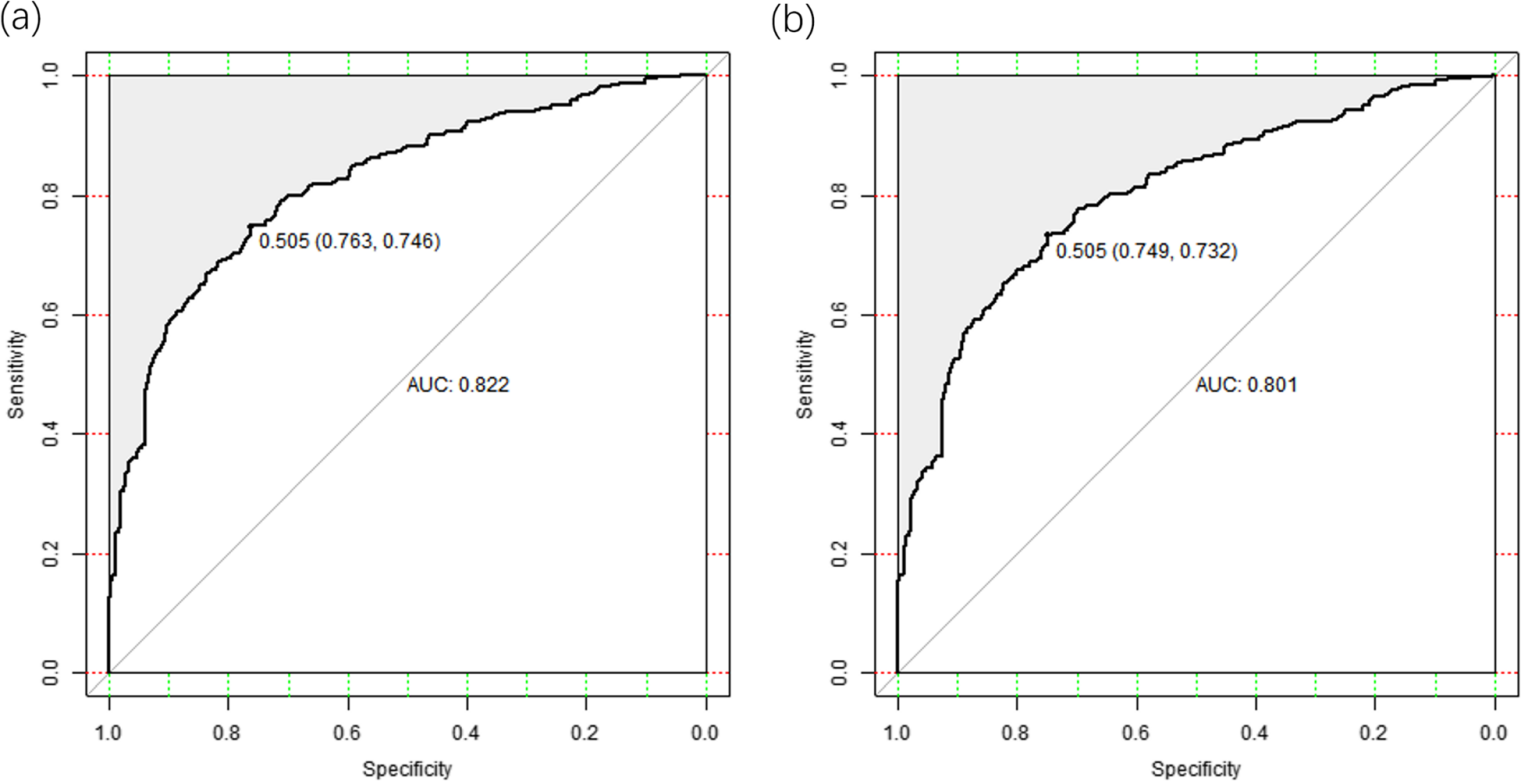
ROC curve validation of risk prediction nomogram for AD in the elderly. **a** Training set: optimal threshold: 0.505; corresponding specificity and sensitivity: 0.763, 0.746; **b** Validation set: optimal threshold: 0.505; corresponding specificity and sensitivity: 0.749, 0.732. The black bold line represents the performance of the nomogram in the training set and validation set. The y-axis represents the true positive rate of risk prediction, and the x-axis represents the false positive rate of risk prediction

The ROC curve is used to assess the discriminating ability of the prediction model. For the prediction model, the AUC of the nomogram was 0.822 in the training set and 0.801 in the validation set (Fig. 3), indicating good performance of the model.

**Fig. 4:**
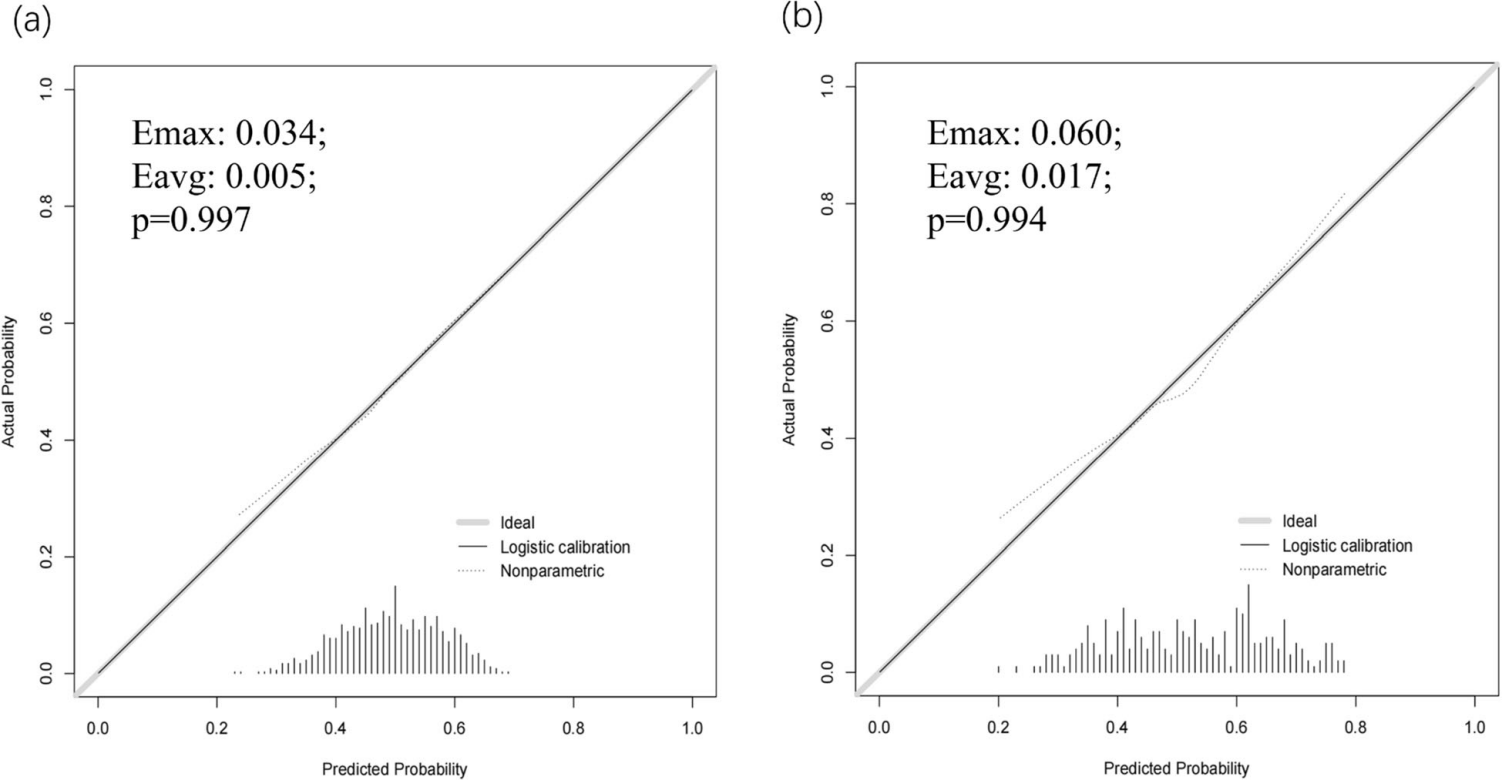
Calibration curve of risk prediction for AD in the elderly. **a** Training set; **b** Validation set. Emax: the maximum offset between the model and the ideal model; Eavg: the minimal offset between the model and the ideal model. *p* > 0.05 indicates passing the calibration test. The black solid line above the x-axis represents sample distribution. The dotted lines on the diagonal represent the perfect prediction of the ideal model, and the solid lines represent the performance of the training set and the validation set. The closer the solid line is to the dotted line, the better the predictive effect. The y-axis represents the actual diagnosed cases of AD, and the x-axis represents the predicted risk of AD

Calibration chart and Hosmer-Lemeshow test were used to calibrate the prediction model. It could be seen from the calibration curve that the prediction model had a good fit with the validation set. Hosmer-Lemeshow test demonstrated that the predicted probability was highly consistent with the actual probability (training set, *p* = 0.997; validation set, *p* = 0.994) (Fig. 4).

**Fig. 5:**
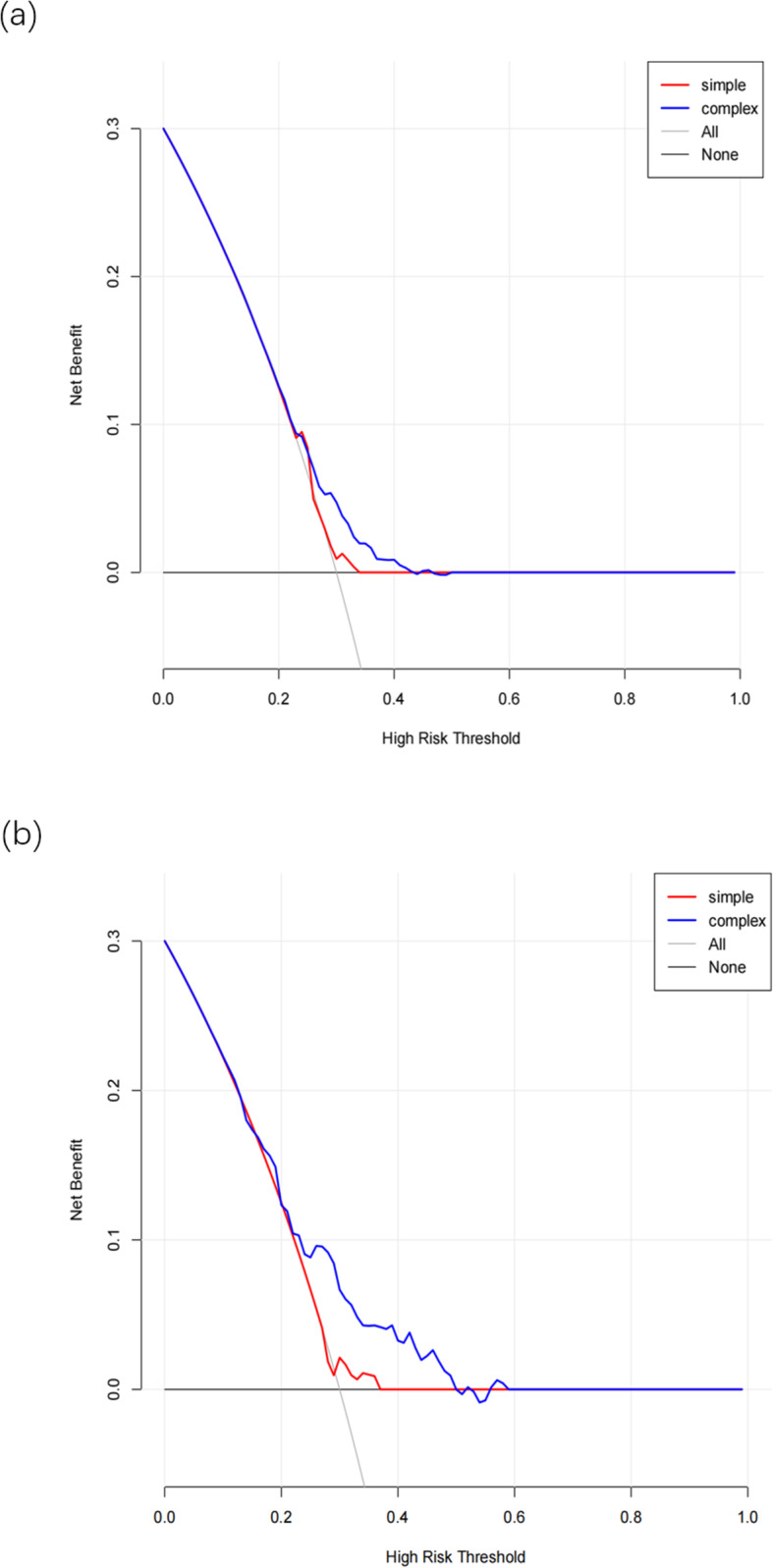
DCA of risk prediction nomogram for AD in the elderly. **a** Training set; **b** Validation set. The black solid line represents the assumption that none of the participants have AD, and gray solid line represents the assumption that all of the participants have AD. The blue thick solid line represents the composited model, combined with sex, age, economic status, health status, lifestyle and genetic risk as prediction methods, and developing AD as the result. The red thick solid line represents a simple model with only a single risk factor included. The y-axis is net benefit, and the x-axis is threshold probability

DCA results exhibited that the threshold probabilities of training set and validation set in the prediction model were 30–40% and 30–42%, respectively (Fig. 5), indicating that the model had good application value.

## Discussion

In this study, we constructed a risk prediction model for AD in the elderly. Sex, age, economic status, health status, lifestyle and genetic risk are independent risk factors for AD in the elderly. Age is our first consideration. Since the majority of AD onset occurs over 60 years old, our study was also targeted at the elderly population aged ≥60. Age is an important risk factor for developing AD. Older age indicates higher risk of developing AD, and age has the greatest impact on advanced dementia compared to other factors [28]. A study suggests that sex difference is another important factor for AD, which may involve the secretion of female hormones [29]. The latest report shows that the bone cell-derived hormone osteocalcin (OCN) plays a key role in cognition [30]. OCN levels are associated with bone density and bone conversion, and therefore are highly affected by changes associated with menopause, increasing risk of disease in menopausal women [30]. All of these studies suggest that women are at greater risk of developing dementia in old age, which is consistent with our risk prediction model.

Previous epidemiological studies on lifestyle and dementia have considered diet [31], physical activity [32] and participation in cognitive activities [33] as risk factors. Another two prospective cohort studies of the elderly have linked a healthy lifestyle with a reduced risk of AD [34]. Specifically, the risk of developing AD of the elderly who also adhere to four or five healthy behaviors (high-quality diet, participation in cognitive activities, regular physical activity, light to moderate alcohol and non-smoking) is 60% lower compared with that of people who have none or only one healthy behavior. In this study, participants were rated on their adherence to a healthy lifestyle to predict their risk of disease. In addition, the patient’s own health is also an important factor to be considered. Multiple studies have shown that hypertension increases the risk of cognitive impairment [35] and stroke, of which stroke has been identified as an independent risk factor for dementia [36]. Similarly, elevated glucose can decrease cognitive function and increase the risk of AD [37]. Our study focused on predicting the risk of AD by targeting midlife hypertension, diabetes, herpesvirus infection, stroke, traumatic brain injury, and depression. Notably, a number of studies have also shown a link between adverse childhood experiences, psychiatric symptoms and dementia. A large cohort study found that older Japanese who had three or more adverse childhood experiences had an increased risk of dementia [38]. Another study suggested that chronic psychosocial stress may exacerbate synaptic dysfunction and cognitive impairment in AD through stress-induced abnormalities in microglial function [39]. In addition, some studies found that symptoms such as anxiety and apathy also increase the risk of AD [40–42]. These factors were ignored in our study, which may have led our model to underestimate the risk of AD.

The risk of AD is associated with a variety of genes, and the APOE on chromosome 19 was the first gene identified to be associated with late-onset AD. Up to now, more than 50 risk gene loci have been screened by using genome-wide association technology, and 11 significant load susceptibility loci have been found, and the potential pathogenic mechanism of AD has been explained in terms of cell pathway, immune response, somatic mutation, epigenetics and other aspects [43]. This study evaluated genetic risk based on the family history of dementia. Remarkably, the economic status of all participants was also taken as a predictor in this study. Several studies have shown a strong link between socioeconomic status in early life and the risk of dementia later, with low socioeconomic status often associated with increased morbidity and mortality [44]. The reason might be that low-income population have less access to health care and engage in unhealthy behaviors (such as smoking, an unhealthy diet, alcohol abuse and lack of exercises) more often.

Based on the results of the above risk factors, it is necessary to develop more models to better identify people with risk of AD. An example is that five potential risk factors for AD were identified by using an extended method of Mendelian randomization (MR) - multivariate MR (MVMR) and MR based on Bayesian model averaging (MR-BMA) [45]. Such high-throughput trials can more accurately reflect risk factors for the disease. Another study found that the Framingham cardiovascular Risk Score (FRS) had significant application in predicting dementia risk, particularly the effects of factors such as age and cardiometabolism [46]. In contrast, we applied the nomogram to AD risk prediction. The risk prediction model is of great value in clinical research due to its convenience in application and high diagnostic performance.

This study still has some limitations. First of all, due to limited funds and manpower, we failed to detect the genetic genes and biochemical indicators of the population. Second, the indicators we eventually included were a broad category that could be subdivided according to existing research results. For example, the intake of deep-sea fish, vegetables and fruits has a large proportion in the diet, and periodontitis, hearing impairment and sleep disorder are also important factors to evaluate the health status. Finally, it is necessary to expand the scope of the study population, including the number of subjects and their region, to improve our model.

To sum up, this study investigated the risk factors for AD in the elderly population, and used the nomogram to construct a model to predict the risk of AD via sex, age, economic status, health status, lifestyle and genetic risk. These risk factors are of great significance for early screening and timely prevention of AD. People can significantly reduce the risk of AD by adopting a healthy lifestyle, such as not smoking, drinking as little as possible or not drinking, having a healthy diet, exercising more, and early treatment of various diseases (diabetes, hypertension, anxiety, depression, etc.). In addition, the indicators of this model are relatively easy to acquire and include major risk factors, which can be widely applied to the risk prediction of AD in the elderly population. Based on the assessment, corresponding measures can be taken to reduce the risk of the disease.

## Supplementary Information

**Additional file 1.**

**Additional file 2.**

## Data Availability

The datasets used and/or analyzed during the current study are available from the corresponding author on reasonable request.

## Abbreviations

AD: Alzheimer’s disease
LASSO: Least Absolute Shrinkage Selection Operator
ROC: Receiver operating characteristic
DCA: Decision curve analysis
MCI: Mild cognitive impairment
CSF: Cerebrospinal fluid
BMI: Body mass index
NIA-AA: National Institute on Aging and the Alzheimer’s Association
AUC: Area under the curve
HLtest.R: Hosmer-Lemeshow test
OCN: Osteocalcin
MR: Mendelian randomization
MVMR: Multivariate MR
MR-BMA: MR based on Bayesian model averaging
FRS: Framingham cardiovascular Risk Score
